# Sex-Based Selectivity of PPARγ Regulation in Th1, Th2, and Th17 Differentiation

**DOI:** 10.3390/ijms17081347

**Published:** 2016-08-18

**Authors:** Hong-Jai Park, Hyeon-Soo Park, Jae-Ung Lee, Alfred L. M. Bothwell, Je-Min Choi

**Affiliations:** 1 Department of Life Science, College of Natural Sciences, Hanyang University, Seoul 04763, Korea; hongjai@hanyang.ac.kr (H.-J.P.); hspark91@hanyang.ac.kr (H.-S.P.); jaeunglee@hanyang.ac.kr (J.-U.L.); 2 Research Institute for Natural Sciences, Hanyang University, Seoul 04763, Korea; 3 Department of Immunobiology, Yale University School of Medicine, New Haven, CT 06520, USA; alfred.bothwell@yale.edu; 4 Center for Neuroscience Imaging Research (CNIR), Institute for Basic Science (IBS), Suwon 16419, Korea

**Keywords:** PPARγ, pioglitazone, effector T cells, estrogen, sex

## Abstract

Peroxisome proliferator-activated receptor gamma (PPARγ) has recently been recognized to regulate adaptive immunity through Th17 differentiation, Treg functions, and T_FH_ responses. However, its role in adaptive immunity and autoimmune disease is still not clear, possibly due to sexual differences. Here, we investigated in vitro treatment study with the PPARγ agonist pioglitazone to compare Th1, Th2, and Th17 differentiation in male and female mouse splenic T cells. Pioglitazone treatment significantly inhibited various effector T cell differentiations including Th1, Th2, and Th17 cells from female naïve T cells, but it selectively reduced IL-17 production in male Th17 differentiation. Interestingly, pioglitazone and estradiol (E2) co-treatment of T cells in males inhibited differentiation of Th1, Th2, and Th17 cells, suggesting a mechanism for the greater sensitivity of PPARγ to ligand treatment in the regulation of effector T cell differentiation in females. Collectively, these results demonstrate that PPARγ selectively inhibits Th17 differentiation only in male T cells and modulates Th1, Th2, and Th17 differentiation in female T cells based on different level of estrogen exposure. Accordingly, PPARγ could be an important immune regulator of sexual differences in adaptive immunity.

## 1. Introduction

Peroxisome proliferator-activated receptor gamma (PPARγ), a nuclear receptor and master regulator of lipid metabolism, has emerged as an important regulator of adaptive immunity [1,2,3,4,5,6,7,8,9]. Its ligands have negative regulatory functions in T cell activation [10], proliferation [11,12], and differentiation [13] to prevent or inhibit disease pathogenesis of autoimmune [13,14,15,16,17,18,19,20] and allergic disease models [21,22,23,24,25].

Treatment of T cells with the PPARγ ligands rosiglitazone, ciglitazone, pioglitazone, and 15d-PGJ2 inhibits T cell proliferation and IL-2 production [11,26,27,28]. Ciglitazone treatment increases survival in graft-versus-host disease (GVHD) by Treg cells expressing PPARγ [29]. Differentiation of Th17 cells is inhibited in mice by pioglitazone, thereby delaying disease onset or ameliorating the clinical features of experimental autoimmune encephalomyelitis (EAE) [13]. We previously reported that pioglitazone treatment inhibits human allogenic T cell responses in arterial grafts [12]. PPARγ ligands ciglitazone, rosiglitazone, and pioglitazone also effectively inhibited allergic inflammation in a mouse model of asthma through up-regulation of PTEN [21,22].

PPARγ-deficient T cell animal studies have demonstrated that PPARγ-deficient Treg cells show an impaired ability to regulate effector T cell functions, leading to the development of colitis [14]. More recently, PPARγ-deficient Treg cells displayed impaired migration ability into visceral adipose tissue [30], supporting the influence of PPARγ on Treg functions. In addition, PPARγ selectively inhibits Th17 differentiation to ameliorate EAE [13]. We recently demonstrated that PPARγ acts as a negative regulator in the differentiation of follicular helper T (T_FH_) cells and germinal center (GC) formation by controlling IL-21 and Bcl-6 expression to prevent autoimmunity [31]. Overall, PPARγ plays diverse roles in the regulation of effector T cell functions and autoimmune or allergic diseases.

However, it was suggested that PPARγ is required for the development of colitis in a lymphopenic environment due to the increased apoptosis of PPARγ-deficient T cells [32]. Interestingly, we also reported that PPARγ-deficient T cells in males are more apoptotic, with reduced T_FH_ responses or no significant phenotype in T cell differentiation in vitro, while PPARγ-deficient T cells in females are more easily activated and differentiate into Th1, Th2, Th17, and T_FH_ cells [31].

Given the discrepancies observed in previous studies of PPARγ roles in effector T cells, we hypothesized that PPARγ activation during T cell activation and differentiation varies by sex. Here, we investigated the impact of PPARγ ligand pioglitazone treatment on Th1, Th2, and Th17 differentiation in male and female T cells. We found that pioglitazone treatment inhibited lineage-specific cytokine production in Th1, Th2, and Th17 cells in females and selectively inhibited IL-17 production in Th17 cells in males. These results suggest variable roles by sex for PPARγ in effector T cell differentiation.

## 2. Results

### 2.1. PPARγ Inhibits Th1, Th2, and Th17 Differentiation in Female Mouse Splenic T Cells

**Figure 1:**
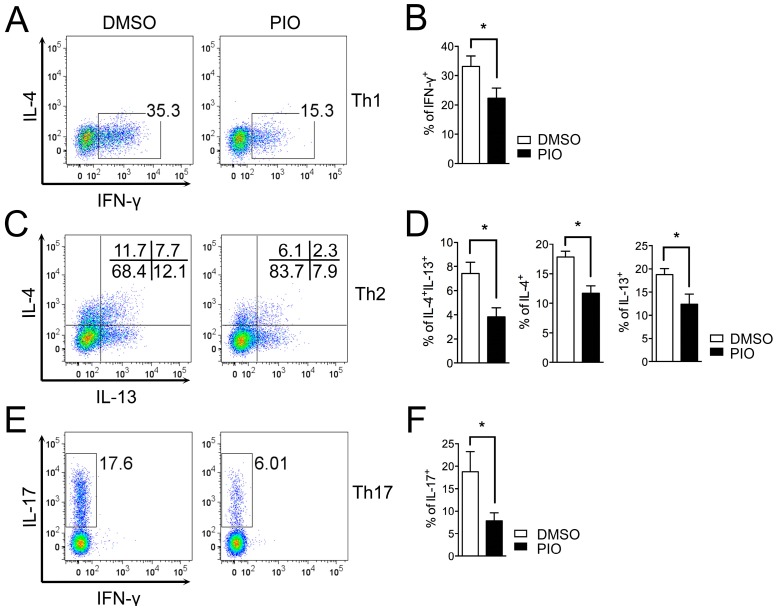
PPARγ activation by pioglitazone treatment inhibits Th1, Th2, and Th17 differentiation in female mouse T cells. MACS-purified CD62L^high^CD44^low^ naïve T cells from the spleens of six- to eight-week-old female C57BL/6 mice were differentiated into Th1 and Th17 cells for three days and Th2 cells for five days in specific cytokine media for T cell–skewing conditions in the presence of DMSO or pioglitazone (20 μM). The proportions of IFN-γ–positive cells in Th1 differentiation (**A**,**B**); IL-4– and IL-13–producing cells in Th2 differentiation (**C**,**D**); and IL-17A–expressing cells in Th17 differentiation (**E**,**F**) were determined by flow cytometry and demonstrated as the dot plots. Values represent mean ± SEM (*n* = 5~6). * *p* < 0.05.

**Figure 2:**
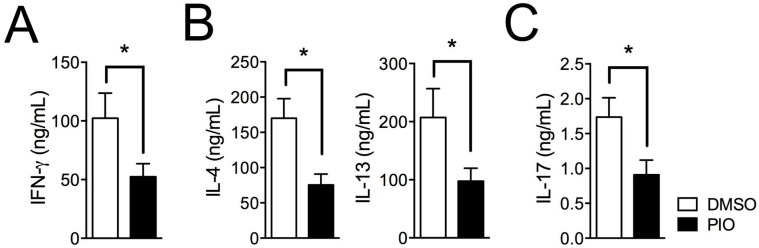
Cytokine production in female mouse Th1, Th2, and Th17 cells is reduced by pioglitazone treatment. The accumulated production of IFN-γ in Th1 cells (**A**); IL-4 and IL-13 in Th2 cells (**B**); and IL-17A in Th17 cells (**C**) in culture supernatants from female T cells treated with DMSO or pioglitazone was measured by ELISA assay. Values represent mean ± SEM (*n* = 5~6). * *p* < 0.05.

To examine the role of PPARγ in Th1, Th2, and Th17 differentiation in female T cells, we investigated the effect of treatment with the PPARγ ligand pioglitazone on Th1, Th2, and Th17 differentiating cells. MACS-purified CD62L^high^CD44^low^ naive T cells from six- to eight-week-old female C57BL/6 mice were differentiated into Th1, Th2, and Th17 cells using specific cytokine media for T cell–skewing conditions with or without treatment with 20 μM pioglitazone. Lineage-specific cytokines were examined by intracellular cytokine staining, and the frequencies of cytokine-expressing cells were analyzed by flow cytometry. Pioglitazone treatment reduced the proportion of IFN-γ–secreting cells in Th1 differentiation (Figure 1A,B), IL-4– and IL-13–expressing cells in Th2 differentiation (Figure 1C,D), and IL-17A–producing cells in Th17 differentiation (Figure 1E,F) compared to DMSO-treated cells. Pioglitazone was not effective in PPARγ-deficient T cells, suggesting that the inhibitory effect was PPARγ-dependent (Supplementary Materials Figure S1). Accumulated cytokine expression in culture supernatants measured by specific ELISA assays demonstrated that IFN-γ in Th1 cells (Figure 2A), IL-4 and IL-13 in Th2 cells (Figure 2B), and IL-17A in Th17 cells (Figure 2C) were significantly reduced by pioglitazone treatment compared to the control group treated with DMSO. These results indicate that the PPARγ agonist pioglitazone can inhibit differentiation of female naïve T cells into effector T cells, including Th1, Th2, and Th17, without specificity.

### 2.2. PPARγ Selectively Inhibits Th17 Differentiation in Male Mouse Splenic T Cells

**Figure 3:**
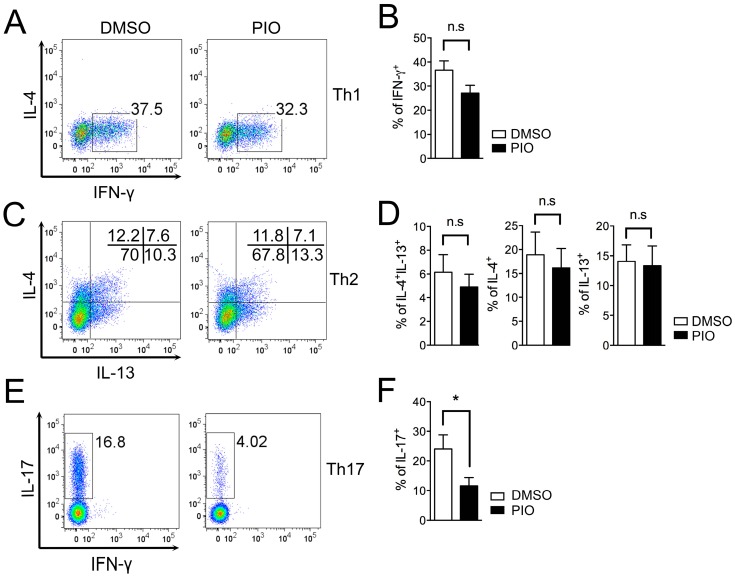
PPARγ activation by pioglitazone treatment selectively inhibits Th17 differentiation in male mouse T cells. MACS-purified CD62L^high^CD44^low^ naïve T cells from the spleens of six- to eight-week-old male wild-type C57BL/6 mice were skewed into Th1 and Th17 cells for three days and Th2 cells for five days. The differentiated effector T cells were treated with DMSO or pioglitazone (20 μM), and the frequencies of IFN-γ–secreting cells in Th1 differentiation (**A**,**B**); IL-4– and IL-13–producing cells in Th2 differentiation (**C**,**D**); and IL-17A–positive cells in Th17 differentiation (**E**,**F**) were determined by flow cytometry and represented as the dot plots. Values represent mean ± SEM (*n* = 4~5). * *p* < 0.05; n.s: non-significant.

**Figure 4:**
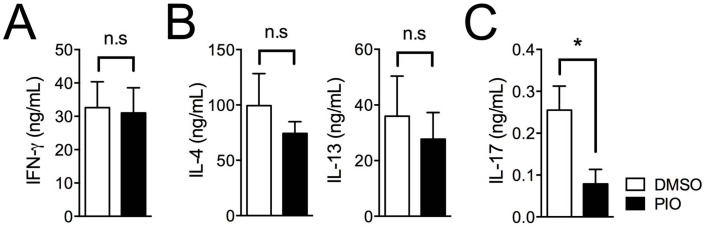
Selective inhibition of IL-17 production in Th17 cells from male mouse T cells by pioglitazone treatment. Accumulated cytokine expression of IFN-γ in Th1 cells (**A**); IL-4 and IL-13 in Th2 cells (**B**); and IL-17A in Th17 cells (**C**) in cultured supernatants from male T cells treated with DMSO or pioglitazone (20 μM) was measured by ELISA. Values represent mean ± SEM (*n* = 4~5). * *p* < 0.05; n.s: non-significant.

While our results demonstrate a potent effect of pioglitazone in effector T cell differentiation, such treatment has previously shown selective inhibition of Th17 cells [13]. In addition, we recently reported sex-based differences in the effects of pioglitazone treatment on T_FH_ cell responses [33]. Thus, we hypothesized that there could be also sex-based differences in the effect of pioglitazone on regulation of Th1, Th2, and Th17 differentiation. To address this question, T cell differentiation experiments were carried out with naive T cells from six- to eight-week-old male C57BL/6 mice. As shown in Figure 3, pioglitazone treatment of T cells from males selectively reduced the frequency of IL-17A–expressing cells in Th17 differentiation (Figure 3E,F) without any effect on IFN-γ–positive cells in Th1 differentiation (Figure 3A,B) or IL-4– and IL-13–producing cells in Th2 differentiation (Figure 3C,D) compared to DMSO-treated cells. Accumulated cytokine production in culture supernatants was analyzed with cytokine-specific ELISA assays, revealing that only IL-17A production in Th17 cells from males (Figure 4C) was inhibited by pioglitazone treatment; production of other cytokines, including IFN-γ in Th1 cells (Figure 4A) and IL-4 and IL-13 in Th2 cells (Figure 4B), was not altered by pioglitazone treatment compared to the vehicle control group. Collectively, these data demonstrate that pioglitazone selectively inhibits the differentiation of Th17 cells in male T cells, while more strongly regulating effector T cells, including Th1, Th2, and Th17 cells in female T cells.

### 2.3. Pioglitazone and Estradiol Co-Treatment Inhibits Th1, Th2, and Th17 Differentiation in Male Mouse Splenic T Cells

**Figure 5:**
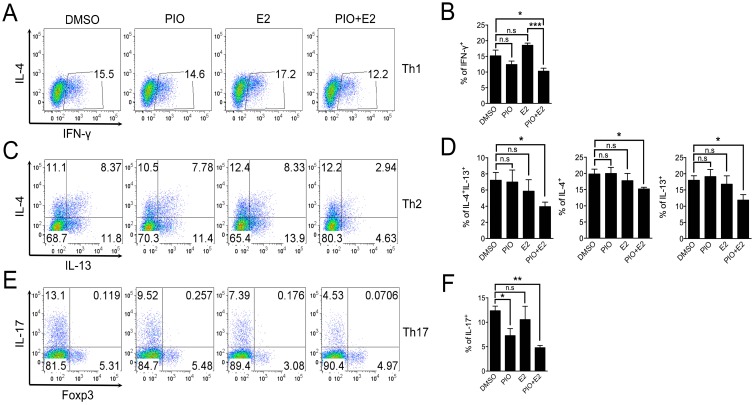
Pioglitazone and estradiol co-treatment inhibits Th1, Th2, and Th17 differentiation in male T cells. CD62L^high^CD44^low^ naïve T cells were purified by magnetic-activated cell sorting (MACS) from the spleens of six- to eight-week-old male wild-type C57BL/6 mice and were differentiated into Th1 and Th17 cells for three days and Th2 cells for five days under lineage-specific skewing conditions with DMSO, pioglitazone (20 μM), E2 (5 nM), or pioglitazone (20 μM) + E2 (5 nM). The proportions of IFN-γ–producing cells in Th1 differentiation (**A**,**B**); IL-4– and IL-13–expressing cells in Th2 differentiation (**C**,**D**) and IL-17A–secreting cells in Th17 differentiation (**E**,**F**) were determined by flow cytometry and depicted as the dot plots. Values represent mean ± SEM (*n* = 4~5). * *p* < 0.05, ** *p* < 0.01, *** *p* < 0.001; n.s: non-significant.

**Figure 6:**
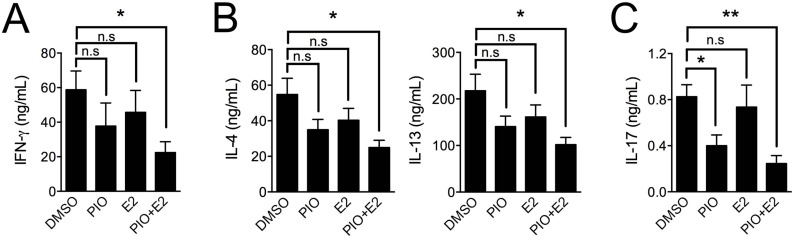
Co-treatment with pioglitazone and estradiol suppresses lineage-specific cytokine production in male Th1, Th2, and Th17 cells. Cultured supernatants from male cells treated with DMSO, pioglitazone (20 μM), estradiol (5 nM), or pioglitazone (20 μM) + estradiol (5 nM) were analyzed by ELISA assay to determine the production of IFN-γ in Th1 cells (**A**); IL-4 and IL-13 in Th2 cells (**B**) and IL-17A in Th17 cells (**C**). Values represent mean ± SEM (*n* = 4~5). * *p* < 0.05, ** *p* < 0.01; n.s: non-significant.

To address the question of whether estradiol treatment helps pioglitazone inhibit Th1 and Th2 cells in addition to Th17 cells of male, as previously demonstrated for their synergy in regulating T_FH_ responses [33], we utilized MACS-purified naïve T cells from six- to eight-week-old male C57BL/6 mice for Th1, Th2, and Th17 differentiation and investigated the effects of co-treatment with 20 μM pioglitazone and 5 nM estradiol. As shown in Figure 5, co-treatment with pioglitazone and estradiol significantly reduced the proportion of IFN-γ–producing cells in Th1 differentiation (Figure 5A,B) and IL-4– and IL-13–expressing cells in Th2 differentiation (Figure 5C,D) compared to the control groups treated with DMSO and estradiol, or pioglitazone, respectively. Co-treatment with pioglitazone and estradiol also effectively inhibited IL-17A–secreting cells in Th17 differentiation compared to DMSO-treated Th17 cells (Figure 5E,F), suggesting that estradiol enhances the negative regulation of pioglitazone on Th1, Th2, and Th17 differentiation. Accumulated cytokine production of IFN-γ in Th1 cells (Figure 6A), IL-4 and IL-13 in Th2 cells (Figure 6B), and IL-17A in Th17 cells (Figure 6C) was also significantly reduced by co-treatment compared to the DMSO-treated control group. These data collectively suggest that estradiol treatment enhances the sensitivity of male effector T cell differentiation to pioglitazone, which might explain the observed sexual differences in pioglitazone effects on Th1, Th2, and Th17 differentiation.

## 3. Discussion

In this study, we observed sex-based differences in the regulation of PPARγ in Th1, Th2, and Th17 differentiation by in vitro pioglitazone treatment of murine naïve CD4 T cells. Differentiation of naïve CD4 T cells into effector T cell subsets was more profoundly affected by PPARγ activation in T cells from females compared to males. Moreover, pioglitizone treatment selectively inhibited the differentiation of Th17 cells only in male T cells, while the addition of estradiol enabled PPARγ activation to regulate Th1 and Th2 differentiation as well. We thus conclude there are sex-based differences in regulatory role of PPARγ in effector T cell differentiation to Th1, Th2, and Th17 cells that are at least partly dependent on the estrogen level.

Ligands for PPARγ are negative regulators of effector T cell responses that ameliorate autoimmune or allergic diseases, including GVHD, EAE, and asthma [13,21,22,25,29]. Recently, the role of PPARγ has been highlighted in T cell responses by utilizing a T cell–specific PPARγ-deficient mouse model, but results have not been conclusive. PPARγ-deficient Treg cells did not suppress effector T cell responses or colitis development in one study [14], while another found that PPARγ was required for the development of colitis in lymphopenic conditions due to the increase of cell death without PPARγ [32]. Correspondingly, PPARγ has been highlighted due to its selective inhibition of Th17 differentiation in regulating EAE disease [13]. However, we previously reported that PPARγ negatively regulates effector T cell differentiation, including Th1, Th2, Th17, Th9, and T_FH_ cells, without specificity [31,33,34].

Here, we explored whether sex-based differences in the role of PPARγ activation might be due to different PPARγ expression levels. We recently reported a very important finding that pioglitazone treatment inhibited T_FH_ induction and GC formation only in females, but not in males, and that estradiol treatment enhanced the effect of pioglitazone in suppressing effector T cell responses in males [33]. In the current study, we observed selective inhibition of pioglitazone on Th17 cells by PPARγ activation in male T cells, and estradiol co-treatment enabled pioglitazone to have the same effect on Th1 and Th2 cells from males that pioglitazone does on T cells from females. These findings raise the possibility of different sensitivities to PPARγ agonist treatment in clinical applications in men and women. There have been previous reports that the PPARγ ligands rosiglitazone and pioglitazone show better sensitivity in women than in men in improving symptoms by decreasing the fasting plasma glucose (FPG) level and increasing the incidence of hypoglycemia in type II diabetes mellitus [35,36]. This finding of sex-based differences in the action of PPARγ in effector T cell responses could help devise sex-specific treatment schemes of human diseases.

Regardless of the sex-dependent roles of PPARγ, pioglitazone treatment suppressed the differentiation of Th17 cells in both male and female T cells, suggesting that PPARγ is an important target to modulate Th17-mediated autoimmune diseases. PPARγ expression also seems to have relevance to multiple sclerosis (MS), with lower expression levels of PPARγ reported in PBMC from MS patients compared to healthy controls [19]. In addition, pioglitazone treatment modestly ameliorates rheumatoid arthritis (RA) activity by preventing bone loss [37] and suppresses plasma levels of cytokines, including TNF-α, IL-1β, and IL-6 [38]. Furthermore, rosiglitazone also reduces glomerular inflammation and autoantibody production in mouse models of systemic lupus erythematosus (SLE) [39], and pioglitazone inhibits the activation and proliferation of effector CD4 T cells from PBMCs of SLE patients [28], suggesting an important role of PPARγ in suppressing autoimmune diseases. Accordingly, PPARγ activation by agonists offers an important strategy for the treatment of autoimmune diseases.

In addition to regulating autoimmune responses by PPARγ agonists, pioglitazone also affects allergic responses. We demonstrated that pioglitazone treatment inhibited female Th2 differentiation by reducing both IL-4 and IL-13 production, which are important for allergic disease pathogenesis. Previously, pioglitazone treatment was shown to effectively suppress allergen-induced bronchial inflammation and airway hyper-responsiveness (AHR) by decreasing IL-4, IL-5, and IL-13 cytokine production and the number of infiltrated eosinophils [22]. Ciglitazone treatment also reduces the production of ovalbumin (OVA)-specific IgE [24], and rosiglitazone treatment increases the production of IL-10 and suppresses the migration of dendritic cells (DCs) to lymph nodes, ameliorating the severity of asthma [23]. Therefore, PPARγ activation by ligands could be also considered for the treatment of allergic diseases such as asthma.

Given that the effects of pioglitazone treatment were more potent on T cells from females, PPARγ ligand treatment might be more effective in regulating disease pathogenesis in females than males. Further study is needed to prove any sex-specific sensitivity of PPARγ-mediated disease regulation. For males, pioglitazone treatment could be a specific inhibitor of Th17 responses for the treatment of autoimmune diseases, and estradiol co-treatment also could be considered for more potent suppression of the T cell response. The synergistic effects of pioglitazone and estradiol in inhibiting male Th1, Th2, and Th17 cells should be further studied in vivo to elucidate the underlying mechanisms.

## 4. Experimental Section

### 4.1. Mice

C57BL/6 wild-type mice were purchased from Orient Bio (Seongnam, Korea). CD4-specific PPARγ-knockout mice (CD4-PPARγ^KO^) were generated by crossing CD4-Cre transgenic mice with PPARγ^fl/fl^ mice. Mice were maintained at Hanyang University mouse facilities under pathogen-free conditions. All procedures regarding isolating splenocytes from the mice and related experiments were approved by the Animal Experimentation Ethics Committee of Hanyang University (2015-0014A (2 February 2015~7 August 2017)) and the experiments were performed according to the guidelines of the Institutional Animal Care and Use Committees (IACUC) of Hanyang University.

### 4.2. Naive T Cell Isolation

Spleens and lymph nodes were isolated from six- to eight-week-old male and female mice. Spleens were incubated with RBC lysis buffer at room temperature for 1 min. The single-cell suspension was incubated with naive CD4 T cell biotin-antibody cocktail at 4 °C for 5 min, and then anti-biotin and CD44 microbeads were added and incubated at 4 °C for 10 min. Naive CD4^+^ T cells were isolated from the mixture by negative selection using a naive CD4^+^ T cell isolation kit and magnetic cell separator (Miltenyi Biotech, Bergisch Gladbach, Germany) according to the manufacturer’s instructions. Purity of MACS-purified naïve T cells was determined by staining with CD62L-FITC, CD44-PE (eBioscience, San Diego, CA, USA), and CD4-PerCP-Cy5.5 (Biolegend, San Diego, CA, USA) fluorescently labeled antibodies. The proportion of CD62L^high^CD44^low^ gated on CD4-positive cells was usually higher than 96%–98%.

### 4.3. T Cell Differentiation

MACS-purified naïve T cells were differentiated for three days under Th1- and Th17-skewing conditions and for five days under Th2-skewing conditions in the presence of pioglitazone and/or estradiol. The purified cells were seeded at 2.5 × 10^5^/well onto a 96-well plate (BD Falcon, San Jose, CA, USA) coated with 2 μg/mL of anti-CD3 and anti-CD28 antibodies (BD Bioscience, San Jose, CA, USA). The following conditions were used to skew differentiation: for Th1 cells, α-IL-4 5 μg/mL, IL-2 50 U/mL, IL-12 1 ng/mL; for Th2 cells, α-IFN-γ 5 μg/mL, IL-2 50 U/mL, IL-4 30 ng/mL; and for Th17 cells, α-IFN-γ and α-IL-4 5 μg/mL each, IL-6 30 ng/mL, TGF-β 1 ng/mL, IL-23 20 ng/mL, and IL-1β 20 ng/mL. For PPARγ ligand and estradiol treatment, pioglitazone (20 μM, Enzo Life Science, Farmingdale, NY, USA) was added, and the culture incubated for three days (Th1 and Th17 cells) or five days (Th2 cells) in the absence or presence of estradiol (5 nM); DMSO served as a vehicle control.

### 4.4. Flow Cytometry

For intracellular staining, differentiated cells were stained with a Near-IR Live/Dead staining kit (Life Technologies, Carlsbad, CA, USA) at room temperature for 15 min to exclude dead cells. After washing, the cells were stained with anti-mouse CD4-PerCP-Cy5.5 antibody (Biolegend, San Diego, CA, USA) at 4 °C for 10 min for surface staining. The cells were then fixed and permeabilized using a Foxp3 staining kit (eBioscience, San Diego, CA, USA) according to the manufacturer’s protocol. Permeabilized cells were stained with the following anti-mouse antibodies at room temperature for 40 min: IFN-γ-FITC and IL-4-PE for Th1 cells, IL-4-PE and IL-13-Alexa Fluor488 for Th2 cells, and IL-17A-PE and Foxp3-APC for Th17 cells. Intracellular cytokine production was examined using the FACSCanto II system (BD Bioscience, San Jose, CA, USA), and data were analyzed using Flow Jo software ver 9.7.6 (Treestar, Ashland, OR, USA).

### 4.5. ELISA

Cytokine production in cultured supernatant from differentiated T cells was measured using mouse IL-4, IL-13, and IL-17A ELISA Ready-SET-Go kits (eBioscience, San Diego, CA, USA) and IFN-γ ELISA MAX sets (BioLegend, San Diego, CA, USA) according to the manufacturers’ instructions.

### 4.6. Statistical Analysis

Data were analyzed statistically with an unpaired two-tailed Student’s *t*-test using Prism5 (GraphPad, San Diego, CA, USA). *p*-values (*p*) less than 0.05 were considered statistically significant.

## Supplementary Materials

Supplementary materials can be found at www.mdpi.com/1422-0067/17/8/1347/s1.
