# Supplementary material for: Sex-Based Selectivity of PPARγ Regulation in Th1, Th2, and Th17 Differentiation
Source: Int J Mol Sci. 2016 Aug 18;17(8):1347. doi: 10.3390/ijms17081347 (PMC5000743; doi:10.3390/ijms17081347)
Supplement: Supplementary file 1 [file ijms-17-01347-s001.pdf]

Supplementary Materials: Sex-Based Selectivity of PPARγ Regulation in Th1, Th2, and Th17 Differentiation

Hong-Jai Park, Hyeon-Soo Park, Jae-Ung Lee, Alfred L. M. Bothwell and Je-Min Choi

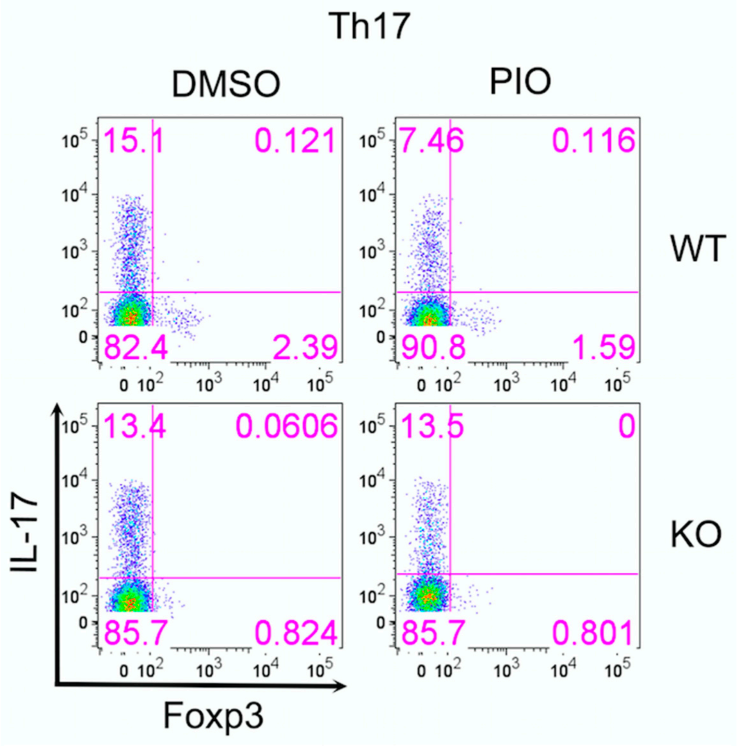

Figure S1. Inhibition of Th17 differentiation by pioglitazone treatment is PPARγ-dependent. Naïve T cells from the spleens of CD4-PPARγ^{KO} and littermate control wild-type mice were differentiated into Th17 cells in the presence of pioglitazone (20 μM) or DMSO. Three days after differentiation, intracellular IL-17 and Foxp3 were evaluated by flow cytometry.
